# How do cancer clinicians perceive real-world data and the evidence derived therefrom? Findings from an international survey of the European Organisation for Research and Treatment of Cancer

**DOI:** 10.3389/fphar.2022.969778

**Published:** 2022-08-24

**Authors:** Robbe Saesen, Georgios Kantidakis, Ann Marinus, Denis Lacombe, Isabelle Huys

**Affiliations:** ^1^ European Organisation for Research and Treatment of Cancer (EORTC), Brussels, Belgium; ^2^ Clinical Pharmacology and Pharmacotherapy Research Unit, Department of Pharmaceutical and Pharmacological Sciences, KU Leuven, Leuven, Belgium

**Keywords:** real-world evidence, real-world data, oncology, cancer, survey, clinicians, randomized controlled trials, Europe

## Abstract

**Background:** The role of real-world evidence (RWE) in the development of anticancer therapies has been gradually growing over time. Regulators, payers and health technology assessment agencies, spurred by the rise of the precision medicine model, are increasingly incorporating RWE into their decision-making regarding the authorization and reimbursement of novel antineoplastic treatments. However, it remains unclear how this trend is viewed by clinicians in the field. This study aimed to investigate the opinions of these stakeholders with respect to RWE and its suitability for informing regulatory, reimbursement-related and clinical decisions in oncology.

**Methods:** An online survey was disseminated to clinicians belonging to the network of the European Organisation for Research and Treatment of Cancer between May and July 2021.

**Results:** In total, 557 clinicians across 30 different countries participated in the survey, representing 13 distinct cancer domains. Despite seeing the methodological challenges associated with its interpretation as difficult to overcome, the respondents mostly (75.0%) perceived RWE positively, and believed such evidence could be relatively strong, depending on the designs and data sources of the studies from which it is produced. Few (4.6%) saw a future expansion of its influence on decision-makers as a negative evolution. Furthermore, nearly all (94.0%) participants were open to the idea of sharing anonymized or pseudonymized electronic health data of their patients with external parties for research purposes. Nevertheless, most clinicians (77.0%) still considered randomized controlled trials (RCTs) to be the gold standard for generating clinical evidence in oncology, and a plurality (49.2%) thought that RWE cannot fully address the knowledge gaps that remain after a new antitumor intervention has entered the market. Moreover, a majority of respondents (50.7%) expressed that they relied more heavily on RCT-derived evidence than on RWE for their own decision-making.

**Conclusion:** While cancer clinicians have positive opinions about RWE and want to contribute to its generation, they also continue to hold RCTs in high regard as sources of actionable evidence.

## Introduction

Before a novel medicine can enter the market, its manufacturer must obtain a marketing authorization from the competent regulatory authorities. To satisfy the stringent evidentiary requirements imposed by regulators such as the European Medicines Agency (EMA) (Scholz, 2015), the US Food and Drug Administration (FDA) (Dabrowska and Thaul, 2018) and the UK Medicines and Healthcare products Regulatory Agency (MHRA) (Criado and Bancsi, 2021), a company needs to present them with, among other things, data derived from clinical trials, which are interventional studies performed in human beings. However, such registrational trials usually have important limitations, including their limited sample size, their relatively short duration and the low external validity of the outcomes they produce (Sherman et al., 2016; Eichler et al., 2018). While they may provide a good indication of how safe the investigational treatment is and how well it works when applied under ideal circumstances (i.e. exactly as intended by its developers), they are not always appropriate predictors of its effects when used by real-life patients (Sherman et al., 2016; Eichler et al., 2018).

As a result, uncertainties often remain with respect to the safety and effectiveness of new therapies at the time of their regulatory approval (Cave et al., 2019). To tackle these uncertainties, additional data are collected in the post-authorization environment. Such data gathered after a therapeutic intervention has been launched onto the market are typically referred to as real-world data (RWD) (GetReal consortium, 2020a). The evidence that arises from the analysis of RWD is called real-world evidence (RWE) (Sherman et al., 2016; GetReal consortium, 2020a). For example, in the European Union, manufacturers of pharmaceutical products are legally obligated to document and report any serious adverse events that are observed in patients taking their drugs in clinical practice as part of the pharmacovigilance legislation (European Medicines Agency, 2022a). This real-world safety information allows for the identification of rare severe side effects and the assessment of long-term risks.

Although RWD and RWE are well-established concepts, there seems to be no consensus on their definition. Makady and others (Makady et al., 2017) found that various different interpretations of these terms exist both in the literature and beyond. Depending on the context, RWD can be used to describe data that originate from non-experimental, non-interventional or non-RCT (i.e. randomized controlled trial) settings. While RWD are frequently understood to be observational in nature, pragmatic clinical trials are also considered by the FDA to be capable of producing RWE (FDA, 2018). Altogether, a multitude of both prospective and retrospective study designs can generate RWE (FDA, 2018; GetReal consortium, 2020b). Common sources of RWD include disease or product registries, electronic health records, insurance claims, pharmacy databases, social media and wearable devices (FDA, 2018; GetReal consortium, 2020c).

In recent years, RWD and RWE have attracted significant attention from stakeholders in the field of drug development^1^. Regulators, payers and health technology assessment (HTA) agencies are increasingly relying on RWE to inform and support their decision-making (Pulini et al., 2021; Flynn et al., 2022). Multiple initiatives have been launched, often with the financial support of the industry, to harness the knowledge contained within RWD (e.g. the GetReal and EHDEN projects undertaken under the auspices of the Innovative Medicines Initiative) (IMI, 2022a; 2022b). However, such efforts have been impeded by the emergence of three main challenges (Cave et al., 2019). Firstly, operational problems have been reported, stemming from the complexities of accessing, sharing and protecting patient data. Hurdles like these are encountered in Europe especially, where the enforcement of the General Data Protection Regulation (GDPR) may exacerbate these issues. Secondly, technical barriers relating to the formatting, linkage and validation of RWD need to be overcome, which requires extensive expertise and optimization of the existing data infrastructure. Lastly, methodological limitations (e.g. selection biases, confounding factors, missing data) have been described, undermining the validity of RWE.

In oncology, the rise of the precision medicine model has curtailed the conduct of large, rigorously designed RCTs (Pregelj et al., 2018). Anticancer treatments are now commonly being approved (whether conditionally or not) based on early-phase clinical data drawn from relatively small, non-randomized, single-arm enrichment studies (Jørgensen, 2021), creating evidence gaps (Kempf et al., 2017) that payers are confronted with when deciding whether or not to reimburse these new therapies (Saesen et al., 2020), which are usually very costly. Some authors (Eichler et al., 2020) see RWD and RWE as potential tools to address these gaps and believe RWE can complement the findings of RCTs, whereas others (Collins et al., 2020) are more skeptical of non-RCT data. Regardless of the beliefs of the latter group, it is likely that the role of RWD and RWE in the antineoplastic drug development process will increase over time (Skovlund et al., 2018), given regulators’ and payers’ growing acceptance of them (Pulini et al., 2021; Flynn et al., 2022).

However, it remains unclear how clinicians who are responsible for treating cancer patients perceive this trend. When making treatment decisions, these healthcare professionals are faced with questions which may not have been tackled yet in previous trials. RWD and RWE could help answer their questions, but doctors may be wary of using them due to the associated methodological constraints. In this study, we aimed to explore the views and perspectives of cancer clinicians regarding RWD and RWE and to gather their opinions about the use of such data and evidence to inform regulatory, reimbursement-related and clinical decision-making in oncology.

## Methods

An online survey consisting of 33 questions (32 multiple-choice and Likert-type questions as well as one open-ended question) divided into five sections was designed (Supplementary Materials) using the SurveyMonkey^®^ platform, based on a scoping review of the relevant literature. This survey was subsequently disseminated to members of the network of the European Organisation for Research and Treatment of Cancer (EORTC), which comprises approximately 2,500 clinicians (including medical oncologists, surgical oncologists, radiation oncologists, dermatologists, hematologists and other specialists that treat cancer patients) working across 750 institutions in 48 countries (EORTC, 2021). Prospective participants received an e-mail containing a link to the questionnaire. Reminder mails were sent out every two weeks until data collection was closed through SurveyMonkey^®^’s built-in reminder function. Responses were recorded between May and July 2021. To ensure that respondents’ answers could not be directly linked back to them, no names, e-mail addresses, IP addresses or institutional affiliations were collected. The study was reviewed and approved by the Ethics Committee Research UZ/KU Leuven (S65201).

The survey data were analyzed descriptively in Excel^®^ and inferentially using IBM^®^ SPSS^®^ Statistics 28.0. Statistical tests were performed post-hoc after checking that the underlying assumptions had been satisfied. For Likert-type questions which asked participants to evaluate multiple items on the same scale, measurement levels were converted to numbers (e.g. “fully disagree” corresponds with 1, “somewhat disagree” with 2, “neither agree nor disagree” with 3, “somewhat agree” with 4 and “fully agree” with 5), allowing for medians, modes and interquartile ranges (IQRs, hereinafter represented by their lower and upper boundaries) to be determined. Friedman and paired-samples Wilcoxon signed-rank tests were then conducted on the numerically transformed data sets in order to verify whether participants perceived the question items differently. One-sample Wilcoxon signed-rank tests were also undertaken to see if the medians leaned significantly towards either end of the answer scale. Where relevant, Jonckheere-Terpstra tests were run to probe whether the survey responses of clinicians with fewer years of experience diverged from those of their more senior colleagues. Sensitivity analyses were carried out to assess the impact of missing data on the outcomes of these tests by imputing absent values with the median. These analyses are not described further in this article since the imputations did not affect the interpretation of the test results. To account for multiplicity, significance levels were adjusted based on Bonferroni corrections.

## Results

In total, 557 clinicians participated in the survey, of whom 500 (89.8%) completed the questionnaire fully and 57 (10.2%) partially, marking a response rate of 22.2% (557/2,505). Since SurveyMonkey^®^ only saves responses when a participant clicks on a specific button to go to the next page, the various sections of the questionnaire had different numbers of respondents.

### Demographic characteristics of survey participants

**FIGURE 1:**
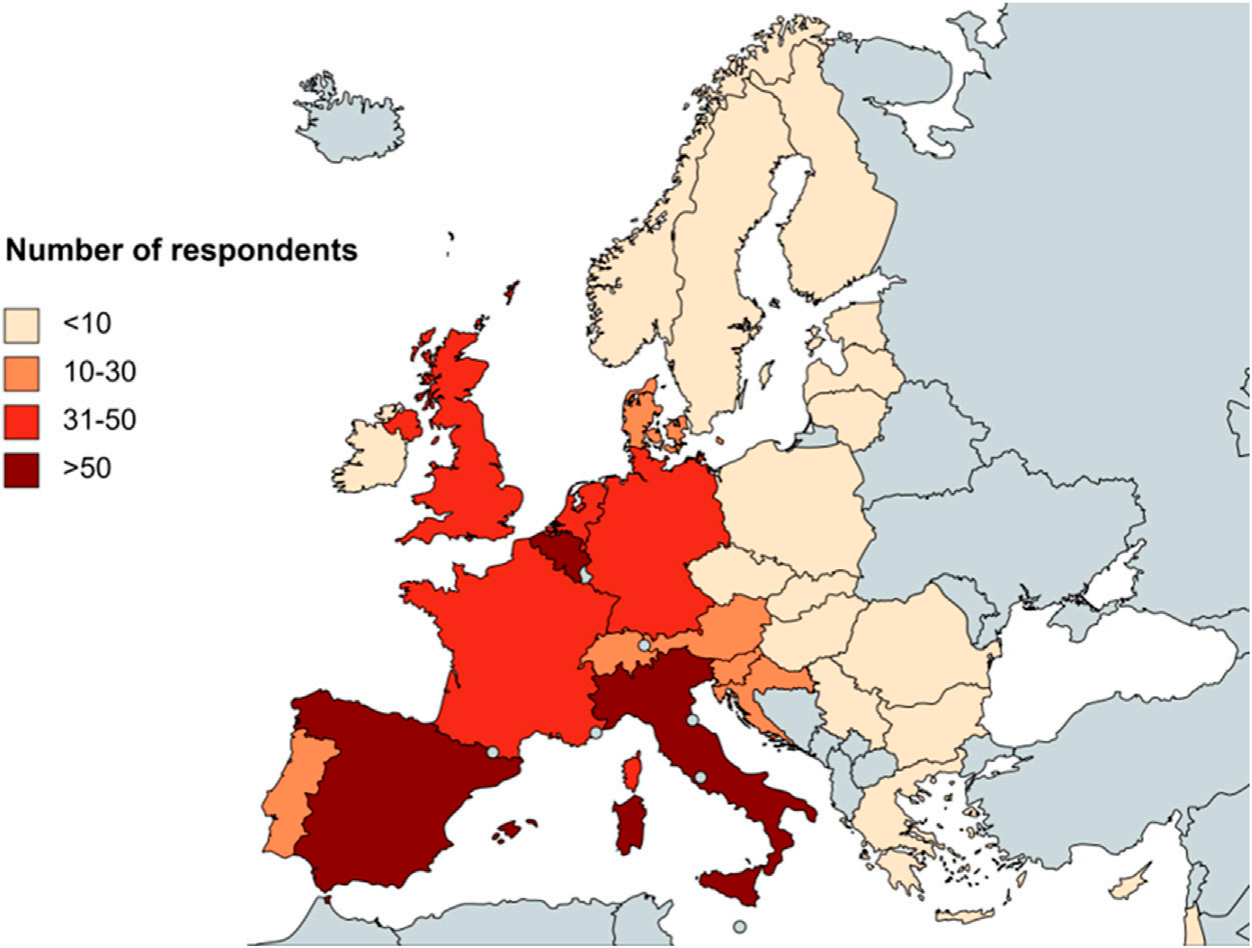
Overview of the number of survey participants per country.

**FIGURE 2:**
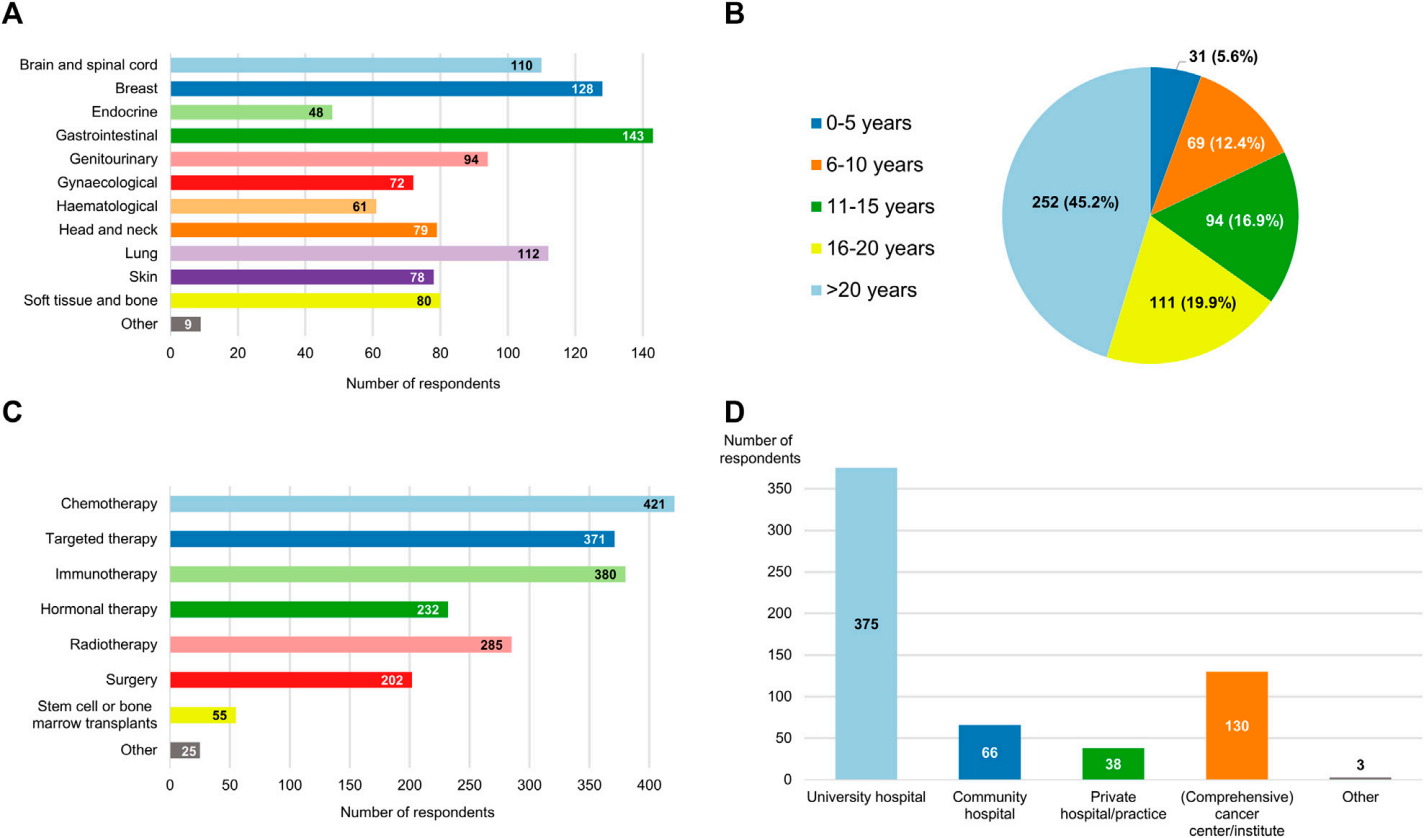
Breakdown of the respondents by **(A)** oncological specialty; **(B)** experience, expressed in years active as a clinician; **(C)** type of anticancer treatments they administer to their patients; and **(D)** type of hospital or institution that employs them. The aggregate numbers reported for **(A**,**C**,**D)** exceed the total number of participants because multiple response options could be selected for these questions.

Clinicians from 30 different countries provided answers to the survey (Figure 1). More than half of the respondents resided in Italy (110/557, 19.7%), Belgium (63/557, 11.3%), Spain (52/557, 9.3%) or France (46/557, 8.3%). 13 distinct oncology domains were represented in the sample, with gastrointestinal cancers (143/557, 25.7%), breast cancers (128/557, 23.0%) and lung cancers (112/557, 20.1%) being the three most common (Figure 2A). A large majority (363/557, 65.2%) of participants were experienced clinicians, having been treating patients for more than 15 years (Figure 2B). In this respect, the treatment modalities most frequently applied by respondents were chemotherapy (421/557, 75.6%), immunotherapy (380/557, 68.2%) and targeted therapy (371/557, 66.6%) (Figure 2C). Nevertheless, the study also saw relatively high levels of participation from clinicians who administered non-pharmacological interventions such as radiotherapy (285/557, 51.2%) and surgery (202/557, 36.3%). In terms of the type of institution the participants were employed at (Figure 2D), approximately two-thirds were working in a university hospital (375/557, 67.3%) and close to a quarter in some kind of specialized cancer center (130/557, 23.3%).

### Understanding of RWD and RWE and their place in the cancer treatment development paradigm

#### Views of the evidentiary criteria used by regulators

**FIGURE 3:**
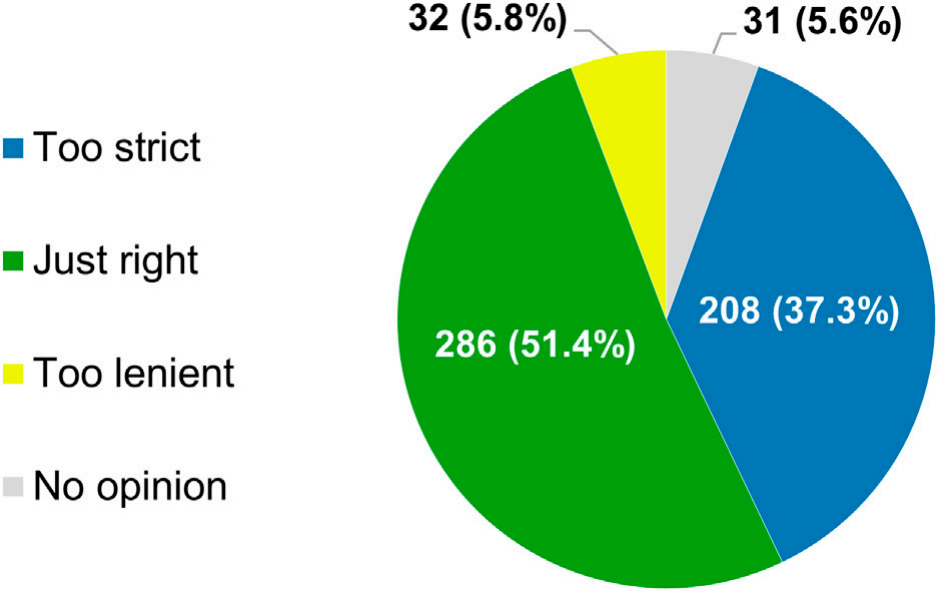
Participants’ views of the evidentiary criteria employed by regulatory authorities in their country or region for allowing new anticancer treatments to enter the market. The percentages shown do not add up to exactly 100% due to rounding.

**FIGURE 4:**
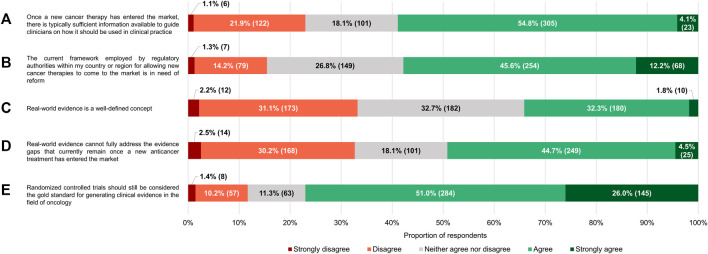
Participants’ level of (dis)agreement with specific statements. The percentages shown for each statement may not add up to exactly 100% due to rounding. The numbers in brackets indicate the absolute number of respondents corresponding with each percentage.

Most of the respondents (286/557, 51.4%) believed that the current framework employed by the regulatory authorities in their country or region for allowing anticancer therapies to come onto the market relies on evidentiary standards that were appropriate for this purpose (Figure 3). Nonetheless, a sizable minority (208/557, 37.3%) were of the opinion that the criteria applied by regulators are too strict. Only 32 participants (5.8%) felt that agencies such as the EMA and the MHRA are too lenient in their decisions to grant marketing authorizations to novel antineoplastic medicines. According to nearly three-quarters of respondents (413/557, 74.2%), the need for rapid patient access to new antitumor treatments and for robust and mature evidence supporting their use in the clinic are equally important factors to consider during the approval process. At the time such interventions are added to the therapeutic armamentarium, there is typically sufficient information available to guide clinicians on how they should be used in clinical practice, 58.9% (328/557) of participants thought (Figure 4A).

Despite their generally positive attitudes towards the existing regulatory standards governing the registration of health technologies in the area of oncology, the majority of respondents (322/557, 57.8%) either agreed or strongly agreed with the notion that the underlying framework requires reform (median of 4 on a scale where 1 means “strongly disagree” and 5 “strongly agree,” which was significantly different from the midpoint with *p* < 0.0005; IQR: 3–4; Figure 4B). Regardless of how long they had been in practice, clinicians wanted to see the current system undergo changes (*p* = 0.939).

#### Perceptions of RWD and RWE

**FIGURE 5:**
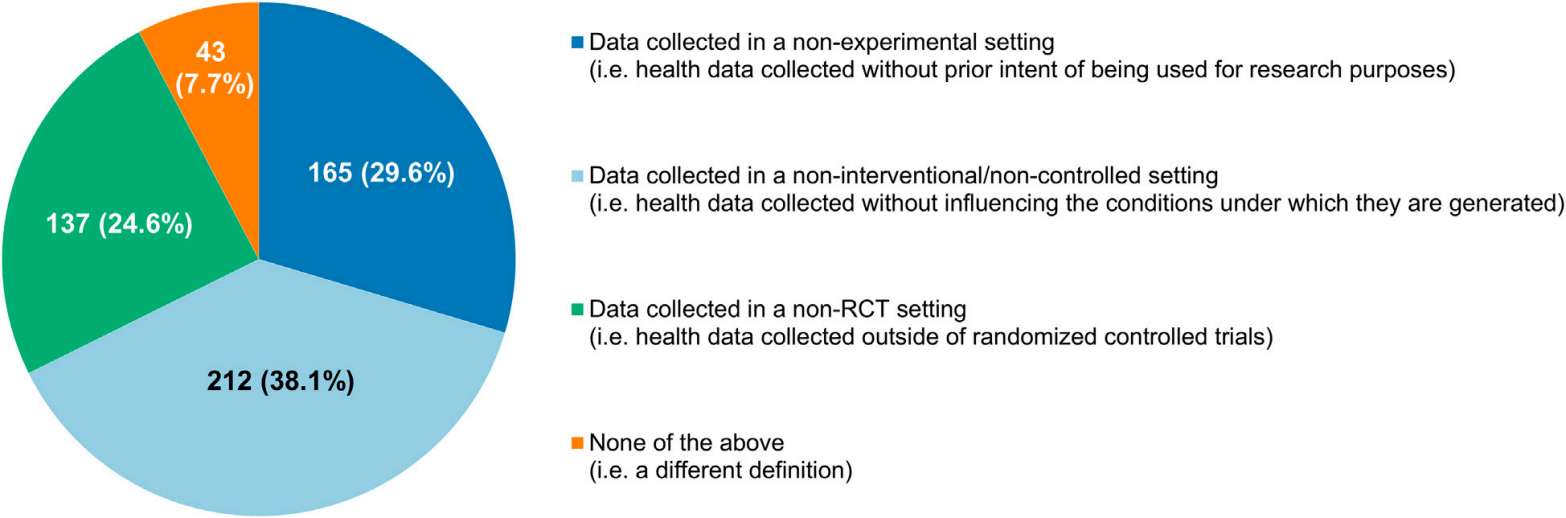
Participants’ understanding of what real-world data are, categorized according to the classification system of Makady et al. (2017).

The proportion of participants who saw RWE as a well-defined concept (190/557, 34.1%) was nearly equal to the percentage of respondents that thought the opposite (185/557, 33.2%), with the remaining 32.7% expressing no opinions one way or the other (Figure 4C). Participants interpreted RWD differently, diverging in their choice of which of the categories of definitions that were documented by Makady et al. (2017) corresponded the closest with their own understanding of the term. A plurality considered RWD to be data collected in a non-interventional/non-controlled setting (212/557, 38.1%), but many also felt that “data collected in a non-experimental setting” (165/557, 29.6%) and “data collected in a non-RCT setting” (137/557, 24.6%) were more fitting descriptions (Figure 5).

**FIGURE 6:**
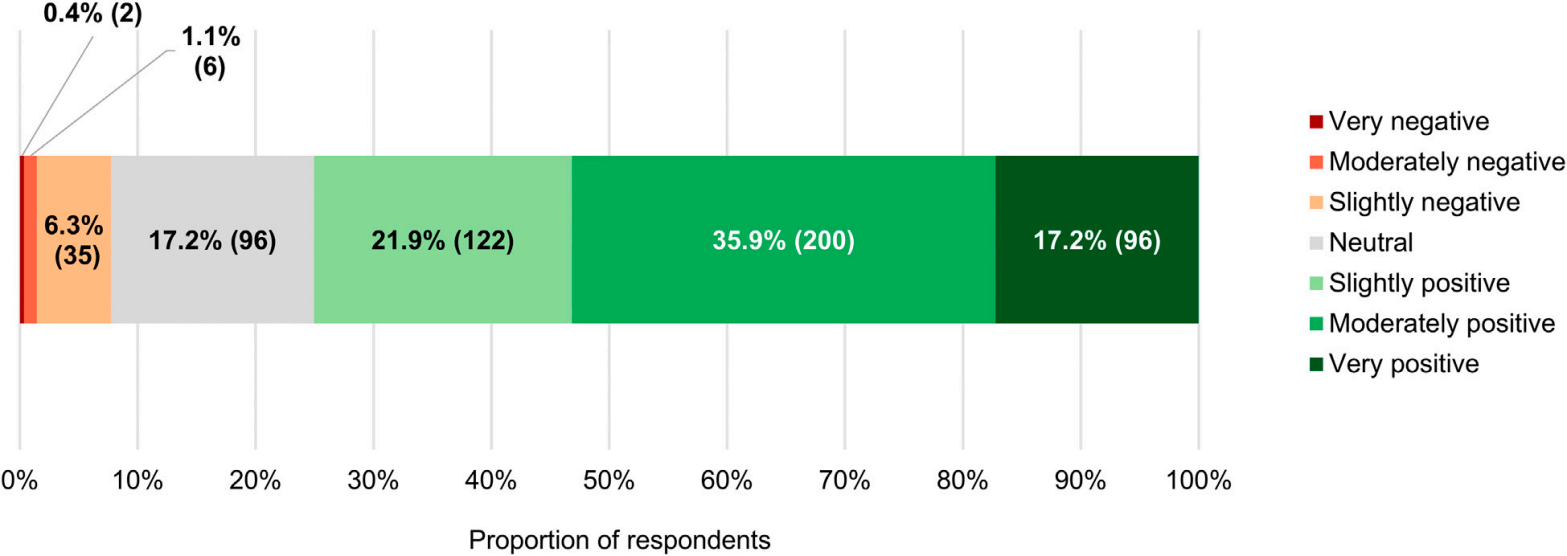
Connotation that the term “real-world evidence” had for participants. The numbers in brackets indicate the absolute number of respondents corresponding with each percentage.

For the vast majority of the clinicians surveyed (418/557, 75.0%), RWE had a slightly (122/557, 21.9%), moderately (200/557, 35.9%) or very (96/557, 17.2%) positive connotation (Figure 6). Just 7.7% (43/557) of respondents had negative perceptions of the term (median of 6 on a 7-point scale where 1 signifies “very negative” and 7 “very positive,” which differed significantly from the midpoint with *p* < 0.0005; IQR: 5–6). In spite of these findings, a near majority of participants (274/557, 49.2%) agreed with the statement that RWE cannot fully address the evidence gaps that currently remain once a new anticancer therapy has entered the market, and only a minority (182/557, 32.7%) believed otherwise (median of 3 on a 5-point scale ranging from 1 or “strongly disagree” to 5 or “strongly agree,” which deviated significantly from the midpoint with *p* < 0.001; IQR: 2–4; Figure 4D). Additionally, more than three-quarters of respondents (429/557, 77.0%) still viewed RCTs as the gold standard for generating clinical evidence in the field of oncology (Figure 4E). The number of years that the clinicians had been treating cancer patients did not influence the connotation RWE had for them (*p* = 0.092), nor did it have an impact on their stance towards RCTs (*p* = 0.844).

### Use and value of RWD and RWE

501 clinicians provided answers for this part of the survey.

#### Impressions of RWD study designs, sources and challenges

**FIGURE 7:**
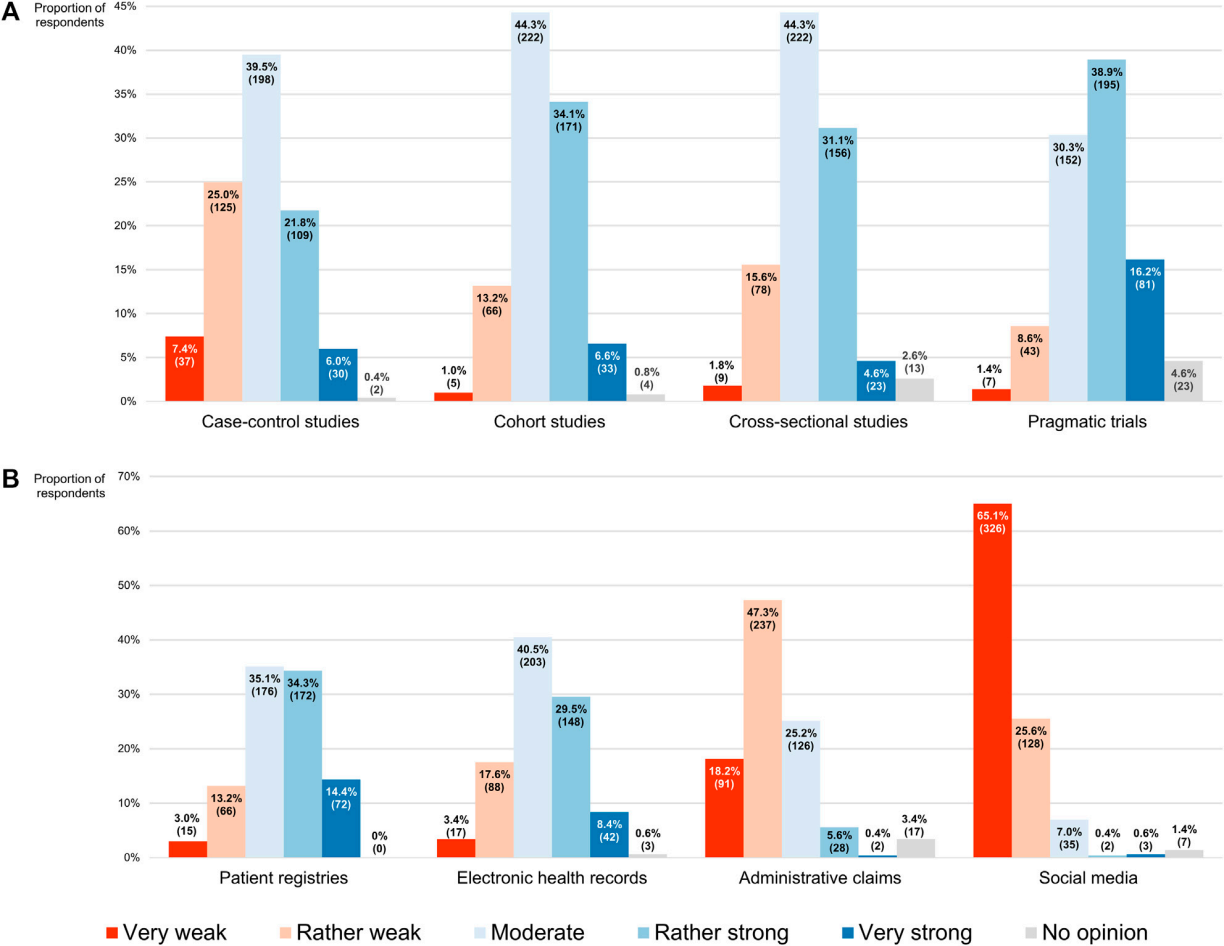
Participants’ perceptions of the strength of the real-world evidence that can be produced by **(A)** different study designs, and **(B)** different sources of real-world data. The percentages shown for each item may not add up to exactly 100% due to rounding. The numbers in brackets indicate the absolute number of respondents corresponding with each percentage.

When participants were asked to evaluate the strength of the evidence produced by observational study designs that are capable of generating RWE on a 5-point scale ranging from “very weak” (numerically equivalent to 1) to “very strong” (numerically equivalent to 5), they most frequently placed them at its midpoint, indicating that they saw case-control, cohort and cross-sectional studies as giving rise to moderately strong evidence (the modal and median scores for all three being 3, with IQRs of 2–4, 3–4 and 3–4, respectively; Figure 7A). On the other hand, pragmatic trials were most commonly considered to deliver rather strong RWE (exhibiting a modal and median score of 4, with an IQR of 3–4). Although the non-interventional methodologies received the same modal and median scores, there was a statistically significant difference in respondents’ perceptions of their evidentiary capacities, with case-control studies ranking lower overall than cross-sectional studies (*p* < 0.001), which in turn scored lower than cohort studies (*p* = 0.006). Being interventional in nature, pragmatic trials preceded all of these (*p* < 0.001).

Similarly, in a subsequent question, participants had to assess the strength of the evidence derived from studies relying on the analysis of RWD that originated from four distinct sources, using the same 5-point scale. Here it was found that the respondents rated patient registries (modal and median score of 3 and IQR of 3–4) significantly higher overall than electronic health records (modal and median score of 3 and IQR of 3–4; *p* < 0.001), which for their part were ranked higher than administrative claims (modal and median score of 2 and IQR of 2–3; *p* < 0.001; Figure 7B). Social media were clearly viewed as a source of RWD that would generate RWE of inferior quality, scoring lowest among the four (modal and median score of 1 and IQR of 1–2; *p* < 0.001).

**FIGURE 8:**
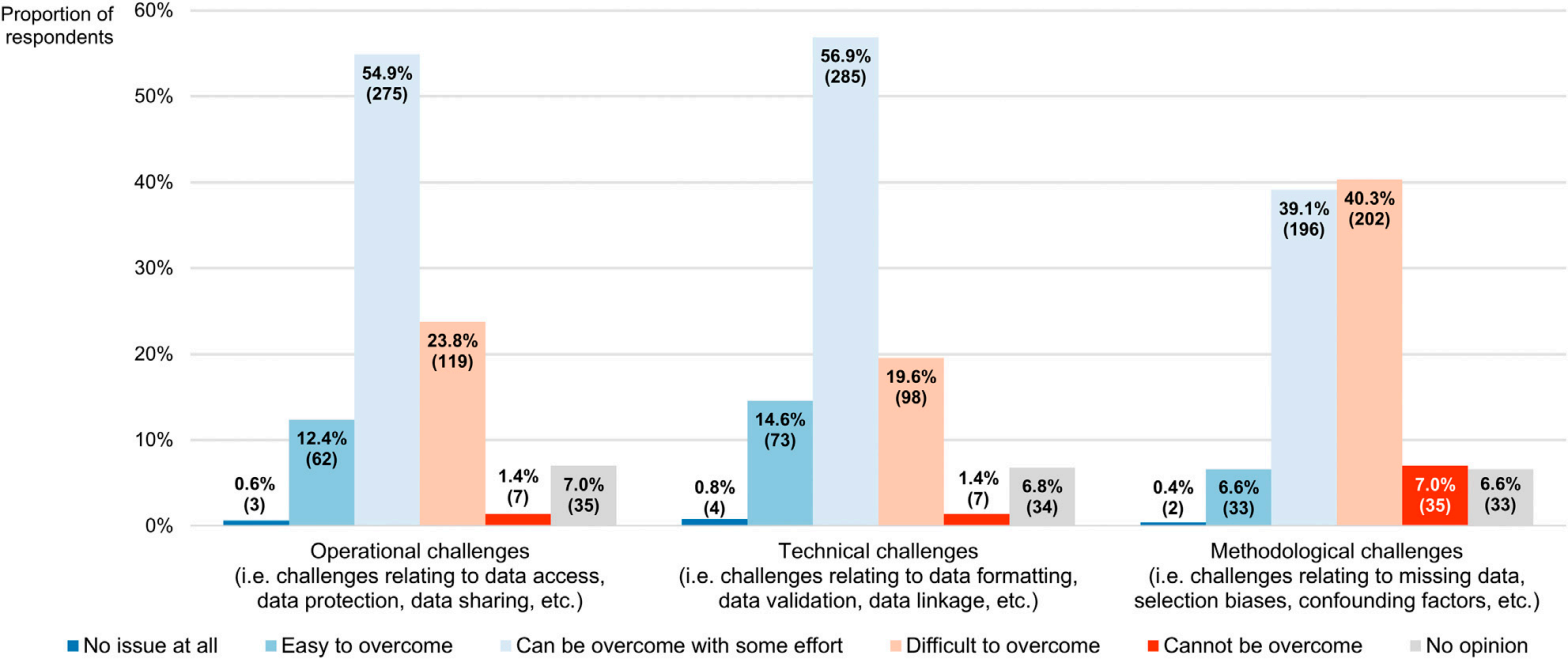
Participants’ perceptions of challenges associated with the collection and use of real-world data. The percentages shown for each challenge may not add up to exactly 100% due to rounding. The numbers in brackets indicate the absolute number of respondents corresponding with each percentage.

Concerning the challenges complicating the collection and use of RWD that have been described in the literature (Cave et al., 2019), most participants believed the operational and technical challenges could be overcome with some effort (54.9% or 275/501 and 56.9% or 285/501, respectively), positioning them at the midpoint of a 5-point scale where the left end (numerically equivalent to 1) meant that they did not interpret them as issues at all and the right end (numerically equivalent to 5) signified that they deemed them impossible to surmount (modal and median scores for both being 3, with IQRs of 3–4 and 3–3, respectively; Figure 8). Respondents regarded the methodological challenges as more difficult to address (modal and median score of 4 and IQR of 3–4), and thus ranked them higher than both the operational barriers (*p* < 0.001) and the technical hurdles (*p* < 0.001), the latter of which received the lowest overall score (*p* = 0.019).

#### Role of RWD and RWE in the decision-making of regulators and payers

**FIGURE 9:**
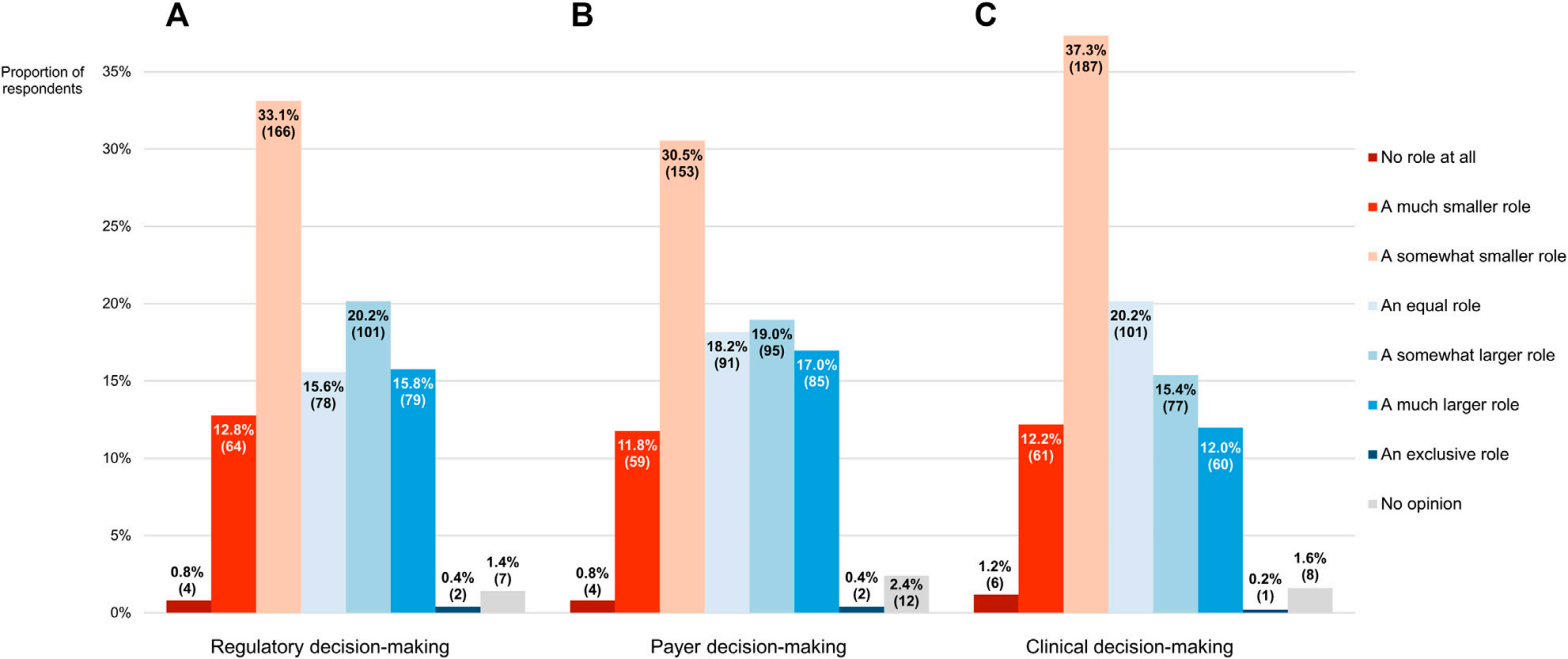
The role that participants thought real-world evidence should play in the decision-making process of **(A)** regulators and **(B)** payers, and **(C)** the role it currently plays in their own decision-making, as compared with evidence derived from randomized controlled trials. The percentages shown for each item may not add up to exactly 100% due to rounding. The numbers in brackets indicate the absolute number of respondents corresponding with each percentage.

As compared with evidence derived from RCTs, RWE should play a smaller role in the decision-making processes of regulators and payers, pluralities of participants thought (46.7% or 234/501 and 43.1% or 216/501, respectively; Figures 9A,B). Nevertheless, more than one-third of respondents (36.3% or 182/501 in both cases) said that the impact of RWE should be greater than that of RCT-generated evidence in these two contexts, with the remaining clinicians either expressing that they should be weighted equally (15.6% or 78/501 and 18.2% or 91/501, respectively) or having no opinion whatsoever (1.4% or 7/501 and 2.4% or 12/501, respectively). On a 7-point numerical scale where a score of 1 corresponded with the view that RWE should not be considered at all and a score of 7 with the stance that no evidence other than RWE should be taken into account, the modal scores for its desired influence on regulatory and reimbursement-related decision-making were 3 and the median scores 4 (IQRs of 3–5). There was no statistically significant difference in the overall scores between the two settings (*p* = 0.068).

When confronted with a scenario where an RCT and a subsequent study relying on the analysis of RWD come to different conclusions regarding the effects of a hypothetical treatment on the overall survival of patients suffering from an unspecified type of cancer, with the former detecting a significant improvement over the standard of care and the latter finding no such benefit, a majority of the participants (303/501, 60.5%) were of the opinion that, depending on the context, the RWE obtained from the second study should potentially be able to lead to modifications of the marketing authorization of the therapy in question, such as a retraction of an approved indication or of the regulatory approval altogether. Likewise, most respondents (265/501, 52.9%) felt that the possibility of RWE engendering revisions of the conditions under which the intervention is reimbursed by healthcare systems, including a reduction in or termination of coverage, should not be excluded in this situation. Moreover, many clinicians believed the outcomes of the RWD study ought to give rise to such policy changes on the part of regulators (122/501, 24.4%) and payers (145/501, 28.9%) regardless of the underlying circumstances.

**FIGURE 10:**
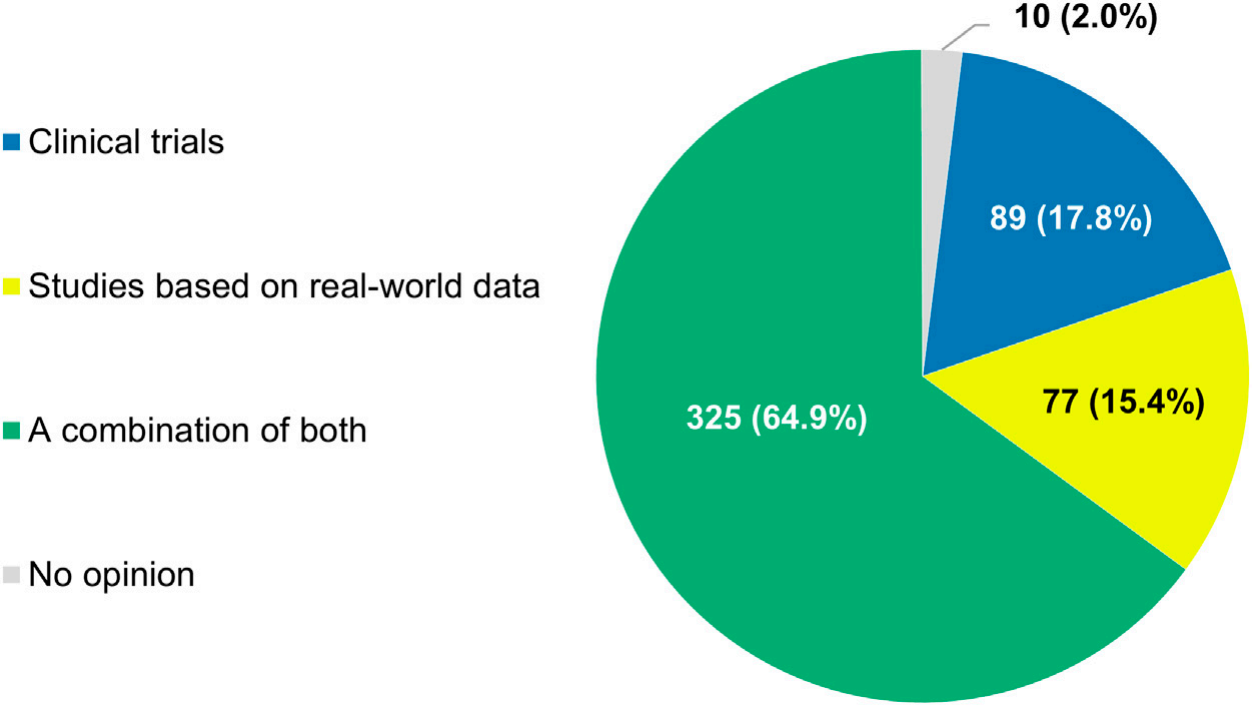
Types of studies that participants thought should be performed when regulators grant marketing authorizations to anticancer treatments on the condition that uncertainties regarding their effectiveness are addressed in the post-approval setting. The percentages shown do not add up to exactly 100% due to rounding.

If the regulatory authorities granted a marketing authorization to an anticancer treatment on the condition that uncertainties regarding its effectiveness were to be addressed by the manufacturer in the post-approval setting, nearly two-thirds of participants (325/501, 64.9%) saw the conduct of a combination of both clinical trials and RWD studies as the ideal approach to satisfy this requirement (Figure 10). Only a minority of respondents argued that either method alone would be sufficient (17.8% or 89/501 and 15.4% or 77/501, respectively).

#### Role of RWD and RWE in the decision-making of clinicians

Upon being asked what role RWE plays in their own decision-making process concerning the therapies they administer to their patients, the clinicians mostly (254/501, 50.7%) conveyed that it was smaller than that of evidence generated from RCTs (Figure 9C). However, a sizeable percentage of respondents (27.5%, 138/501) indicated that they relied more heavily on RWE than on RCT-derived evidence in their daily practice, with another 20.2% (101/501) claiming to depend on both in equal measure. As expressed on a numerical scale ranging from 1 (equivalent to not considering RWE at all) to 7 (equivalent to exclusively considering RWE), the reported influence of RWE on participants’ work in the clinic was characterized by a modal and median score of 3, the latter of which deviated significantly from the midpoint of 4 (*p* < 0.001; IQR: 3–5). There was no divergence in overall scores between more and less experienced clinicians (*p* = 0.884).

The concrete relevance of RWD and RWE for specific clinical activities was evaluated in detail, using a 5-point scale whose left and right ends signified that the importance of RWD for the activity in question was very low (numerically equivalent to 1) and very high (numerically equivalent to 5), respectively. It was observed that respondents viewed RWD as less important for diagnosing patients (modal and median score of 3, IQR of 2–4) than for monitoring them (modal and median score of 4 and IQR of 3–4; *p* < 0.001) or making treatment decisions for them (modal and median score of 4 and IQR of 3–4; *p* < 0.001), both of which were rated similarly from a statistical point of view, with any difference between them deemed to be nonsignificant (*p* = 0.066).

**FIGURE 11:**
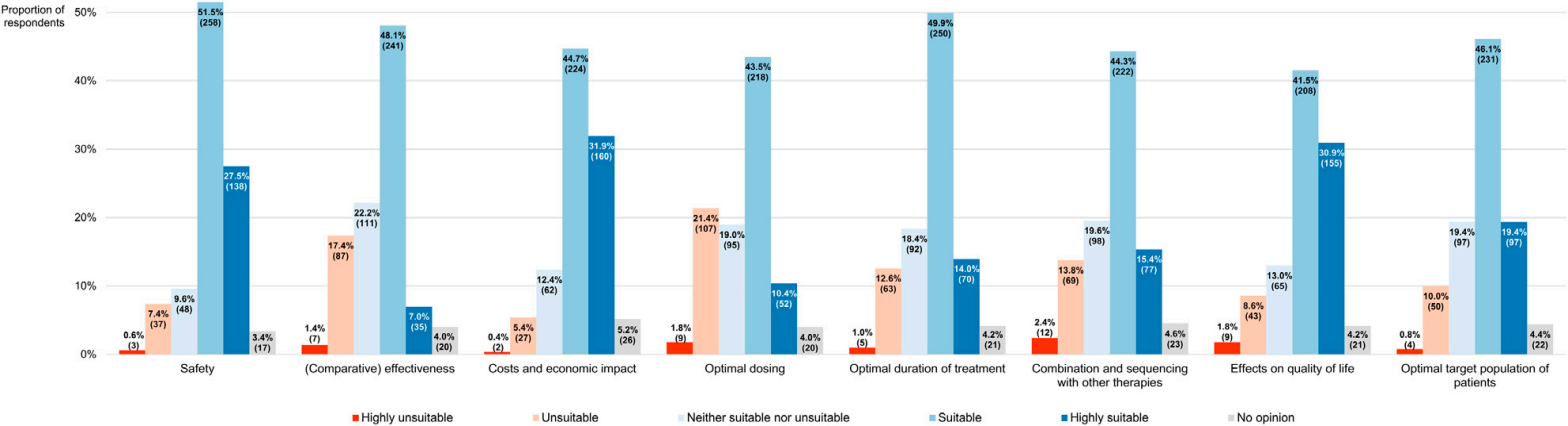
Participants’ views on the suitability of real-world data for addressing different types of uncertainties relating to the adoption of new anticancer therapies into clinical practice. The percentages shown for each uncertainty may not add up to exactly 100% due to rounding. The numbers in brackets indicate the absolute number of respondents corresponding with each percentage.

Participants also had to assess the extent to which RWD can adequately address various uncertainties that are associated with the adoption of antineoplastic interventions into clinical practice, based on a 5-point scale where a score of 1 meant that RWD were highly unsuitable for tackling the uncertainty at issue while a score of 5 indicated that such data were highly suitable for this purpose (Figure 11). In general, the respondents were of the opinion that RWD could serve as a useful tool for answering many outstanding questions relating to the integration of new health technologies into the clinic, as evidenced by the fact that the modal and median scores given by participants were equally high across all the uncertainties they were requested to appraise in terms of RWD’s potential to resolve them, namely 4 (Table 1). Nonetheless, respondents perceived the utility of RWD differently depending on the type of uncertainty (*p* < 0.001), with questions regarding the safety and economic impact of novel therapies receiving a higher overall score than those focusing on their optimal dosing or their effectiveness relative to existing alternatives.

**TABLE 1:** Breakdown of how participants perceived the suitability of RWD for tackling various uncertainties related to the integration of new anticancer treatments into clinical practice, expressed on a scale of 1 (equivalent to highly unsuitable) to 5 (equivalent to highly suitable).

Type of uncertainty	Modal score	Median score	IQR
Safety	4	4	4–5
(Comparative) effectiveness	4	4	3–4
Costs and economic impact	4	4	4–5
Optimal dosing	4	4	3–4
Optimal duration of treatment	4	4	3–4
Combination and sequencing with other therapies	4	4	3–4
Effects on quality of life	4	4	4–5
Optimal target population of patients	4	4	3–4

If the participants were faced with the aforementioned scenario where the results of an RCT and a study based on the analysis of RWD contradict each other, 64.5% (323/501) would not be inclined to trust one over the other without knowing more of the context first. Of the respondents who did have a clear preference outright (177/501, 35.3%), most (118/177, 66.7%) opted to place their confidence in the conclusions drawn from the RCT.

### Experience with and future evolution of RWD and RWE

**FIGURE 12:**
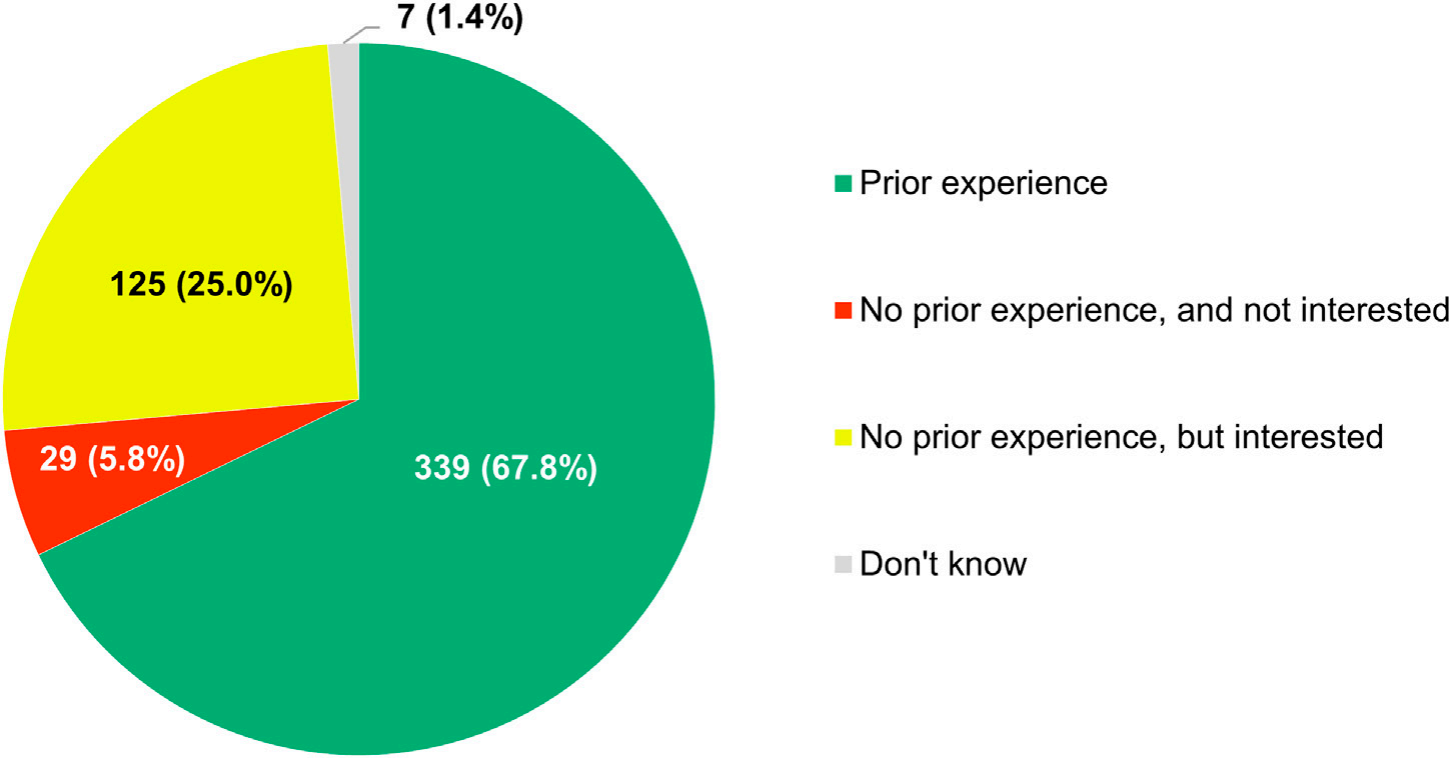
Participants’ reported experience with being involved in studies relying on the analysis of real-world data.

Most of the clinicians who completed this section of the survey (339/500, 67.8%) claimed that they had previously been involved in studies relying on the analysis of RWD (Figure 12). The respondents that had no prior experience with RWE-generating research (154/500, 30.8%) showed strong interest in contributing to it in the future, with 81.2% of them (125/154) expressing willingness to participate therein at some point in time. Furthermore, according to a majority of participants (319/500, 63.8%), there were already studies ongoing at their hospital or institution in which RWD were being collected and analyzed.

**FIGURE 13:**
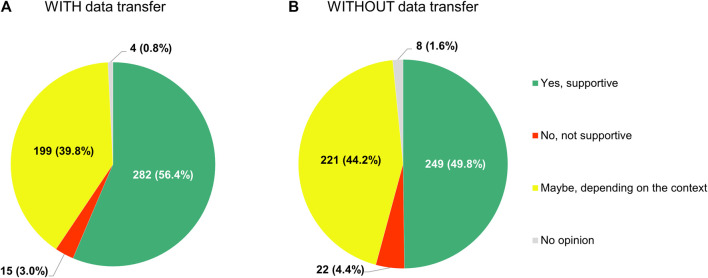
Participants’ support for sharing anonymized or pseudonymized electronic health data of their patients with external parties for research purposes, if **(A)** these data would be transferred to a central, internationally accessible repository, or **(B)** if no such transfer would occur.

Respondents were also open to sharing anonymized or pseudonymized electronic health data of their patients with other researchers inside or outside their country, regardless of whether this would mean that (A) these data would be transferred to an external, internationally accessible, central repository, or, conversely, (B) no such transfer would take place (Figure 13). In the former case (i.e. situation A), 56.4% of participants (282/500) were supportive of the idea and another 39.8% (199/500) refused to exclude it outright, whereas in the latter (i.e. situation B), these numbers were 49.8% (249/500) and 44.2% (221/500) respectively.

**FIGURE 14:**
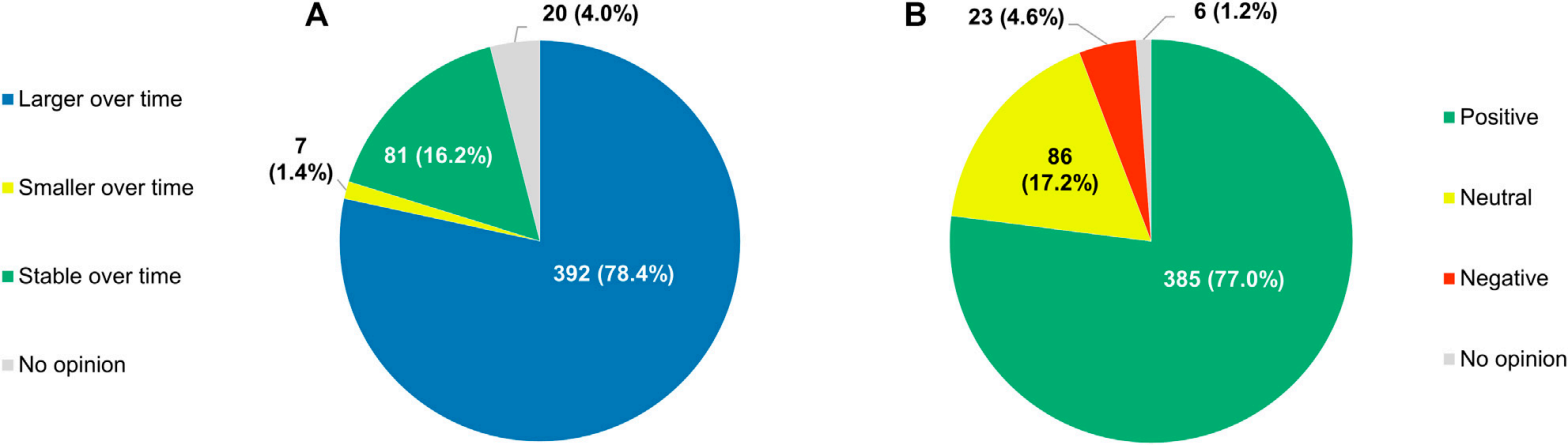
**(A)** Participants’ projections of how the role of real-world evidence in the development of anticancer treatments will evolve over time, and **(B)** their perceptions of a hypothetical growth in its future influence.

With respect to the role that RWE plays in the development of anticancer treatments, 78.4% (392/500) of respondents expected it to increase in the future (Figure 14A). Only a small subset of participants (88/500, 17.6%) did not see RWE becoming more important over time, with 16.2% (81/500) anticipating its influence to remain unchanged and 1.4% (7/500) foreseeing a decline of its significance. If the importance of RWE for developing antitumor therapies were indeed to grow in the years to come, 77.0% (385/500) of the clinicians surveyed would perceive this trend positively, 17.2% (86/500) would have a neutral stance towards it, and just 4.6% (23/500) would view it negatively (Figure 14B).

## Discussion

To our knowledge, this study is the first to provide a comprehensive overview of how European and Israeli cancer clinicians look at RWD and RWE. As the results show, the respondents to this large, international survey generally expressed positive opinions about RWE and displayed openness to the idea of getting involved in studies relying on the analysis of RWD. While they saw the methodological challenges associated with its interpretation as difficult to surmount, participants thought RWE could be relatively strong, as long as the design, the objectives and the data sources of the study from which it is derived are chosen well. Nevertheless, in their view, the role of RCTs in the development of antineoplastic therapies remains indispensable.

Some authors (Kish et al., 2018; Klink et al., 2019) have probed the perspectives of US-based community oncologists regarding RWE, but their findings have been exclusively published in the form of conference abstracts. Kish et al. (2018) investigated the influence of RWE on the treatment-related decision-making of cancer physicians surveyed during two different live meetings. Although 76% of the 122 respondents indicated that they believed RWE is necessary to inform clinical practice, 69% stated that they never or infrequently used the results of RWD studies to guide their therapeutic decisions, and only 21% claimed to have participated in such studies before. This discrepancy between the perceived value of and the practical experience with RWE was not present in our study, potentially because RWE has become more entrenched in the oncology field since 2018, when Kish et al. (2018) presented their data. Additionally, clinicians that are part of the EORTC network are primarily academic oncologists who are probably more engaged with research in general.

Despite their inexperience in this area, community oncologists from the US do appear to be willing to contribute to RWE generation: in their survey of this target group, Klink et al. (2019) observed that 89% of the 59 participants asserted that they were somewhat to very likely to get involved in RWD studies. The respondents to our questionnaire expressed similar levels of enthusiasm with respect to taking part in these types of studies. Klink et al. (2019) also reported that 79% of the physicians in their sample considered RWE to be observational data collected outside clinical trials. This description combines two different categories of definitions for RWD as recorded by Makady et al. (2017) (namely, data collected in a non-interventional/non-controlled setting and data collected in a non-RCT setting), which together represented what most of the clinicians in our study understood to be RWD. It should be noted that the surveys of Kish et al. (2018) and Klink et al. (2019) were small in size and that the external validity of their conclusions may be limited.

Besides these two studies, no relevant research papers exploring the attitudes of cancer physicians towards RWD and RWE could be retrieved from the literature. Outside of the academic sphere, a recent study (COTA Healthcare, 2021) commissioned by a US-based healthcare technology company surveyed practicing oncologists across the country and found that 83% of the 200 respondents believed that RWD is critical for accelerating the development of antitumor treatments. Moreover, 78% felt that RWD should be integrated into clinical trials through the incorporation of design elements such as external control arms. While the results of this commercially oriented study which did not undergo peer review should be taken with caution, these data show that, just like their European and Israeli colleagues, many American cancer clinicians view RWD positively.

Although the attitudes of oncologists towards RWD and RWE have been scarcely studied, the perceptions of other stakeholders regarding these concepts have been documented more extensively, both in the cancer field and beyond. For example, Stahl et al. (2018) sent out questionnaires to health system leaders in the US with oncological expertise and found that 80% of respondents saw aggregated, de-identified RWD as extremely important for guiding physician decision-making relating to complex patient cases. Additionally, 60% of participating health systems were already partaking in RWD sharing collaborations. These numbers demonstrate how the use of RWD to inform clinical practice in oncology was generally accepted, which was reflected in our study as well. It should be stressed though that for this specific purpose, cancer clinicians prefer to rely on data originating from RCTs.

Most of our participants thought RWE had some role to play in the decision-making processes of both regulators and payers, albeit a smaller one than that of RCT-derived evidence. They did not consider RWE to be more or less useful to either stakeholder group, at least in comparison to evidence produced by RCTs. The experts who filled in the survey of Gill et al. (2017) also doubted that the results of RWD studies would ever be weighted similarly to those of RCTs in evaluations of product dossiers. However, they were largely of the opinion that RWE was more likely to be applied for supporting reimbursement-related decisions than for influencing the conditions under which marketing authorizations are granted. The perceived value of RWE for payer decision-making has been examined in other studies (Hampson et al., 2018; Malone et al., 2018; Clausen et al., 2020; Brixner et al., 2021; Sievers et al., 2021), which employed a variety of different qualitative research methodologies. The perspectives of stakeholders regarding the utility of RWE for regulatory assessments have been less frequently characterized in the literature (Sievers et al., 2021).

Villines et al. (2020) distributed a questionnaire to a representative sample of US cardiologists with the aim of investigating their knowledge, awareness and uptake of RWE. The 173 respondents were broadly familiar with RWE and viewed its importance for making treatment decisions as lower than that of RCTs, mirroring the sentiments captured in our survey. Moreover, they displayed more confidence in registries as sources of high-quality RWD than in electronic health records, which they in turn rated higher than administrative claims, placing the lowest levels of trust in social media. In our study, participants ranked these RWD sources in the same order when asked to evaluate them in terms of the strength of the RWE that they could generate. Among the cardiologists who had expressed familiarity with RWE, 23% always or often incorporated such evidence into their decision-making, whereas 48% did so sometimes and 21% claimed to never or rarely rely on RWE (Villines et al., 2020). While our survey did not include a question that would elicit comparable information, only 1.2% of our respondents indicated that they did not base themselves on RWE at all for any of their clinical decisions. In the study of Villines et al. (2020), the main concerns raised by participants with respect to RWE were its methodological shortcomings, relating to the non-randomized nature of RWD studies and the associated risk of confounding. Likewise, methodological hurdles were seen by our participants as the most difficult to overcome challenges complicating the analysis and use of RWD. Overall, the findings of Villines et al. (2020) align closely with ours, suggesting that clinicians across different fields share similar opinions on RWD and RWE.

There was no consensus among the cancer clinicians who provided responses to our survey on which of the categories of RWD definitions identified by Makady et al. (2017) best described such data. A plurality perceived RWD to be data collected in a non-interventional/non-controlled setting, but more than half of the respondents felt that one of the other descriptions they were presented with reflected their understanding of the concept more accurately. There was a clear disconnect between how the term RWD was applied by the sources consulted by Makady et al. (2017) (including scientific and grey literature) and how it was interpreted by our respondents: the category of definition that was encountered most commonly by Makady et al. (2017) (namely, data collected in a non-RCT setting) was the least frequently selected option in our survey (excluding the “none of the above” answers). Furthermore, only about a third of our participants considered RWE to be a well-defined concept. This illustrates the need for formulating standardized, internationally agreed upon definitions for RWD and RWE, so as to avoid confusion and facilitate discussion and collaboration on these topics. Regulators in particular are uniquely positioned to drive any harmonization efforts in this regard, for example through the International Coalition of Medicines Regulatory Authorities.

A majority of the clinicians we surveyed believed that regulatory authorities employ the right evidentiary criteria when assessing whether or not a new anticancer treatment should be able to enter the market. Additionally, respondents generally thought that at the time of market entry, there is typically sufficient information available on how novel antineoplastic therapies should be used in clinical practice. However, simultaneously, most participants were also convinced that the current regulatory framework requires some degree of reform. It remains to be seen what they envisaged this reform would look like exactly. Some clinicians may have wanted regulators to grant marketing authorizations faster by becoming more lenient in their evaluations of product dossiers, given that a significant proportion of respondents expressed the opinion that the standards applied by agencies such as the EMA and the MHRA are too strict. This stance would not be unique to our sample nor uncommon altogether: a 2002 poll showed that 61% of US oncologists at the time agreed with the statement that the FDA was too slow in approving drugs and medical devices (Eastman, 2002). Criticisms (The Wall Street Journal Editorial Board, 2011; Dixon, 2017; Marchand, 2019) of the supposed sluggishness of regulatory review and registration procedures are often motivated by a desire to expedite patients’ access to promising medicines. Nevertheless, this particular motive may not fully explain participants’ eagerness for change, since they largely viewed the need for rapid availability of innovative interventions and for robust and mature data on their efficacy and safety as equally important factors to consider during the approval process. Further research efforts to uncover the specific aspects of the regulatory framework that should be amended according to cancer clinicians are therefore warranted. It should be noted here that the belief that regulators are too conservative in their assessments of antitumor agents clashes with the seeming consensus in the academic literature that they are actually too permissive in this respect, and that the evidentiary bar should be raised in light of, among other issues, the proliferating use of shortcut-to-market schemes (Light and Lexchin, 2015; Mintzes and Vitry, 2019; Schnog et al., 2021). From a regulatory perspective, the drawbacks of these schemes are compensated for by the fact that they enable potentially lifesaving treatments to reach cancer patients with greater speed (Beaver and Pazdur, 2021).

We observed widespread receptiveness among our respondents to the idea of sharing their patients’ anonymized or pseudonymized electronic health data with others inside or outside their country for research purposes, no matter whether that would mean that these data would then be transferred to an external repository or not. This finding demonstrates that, although acquiring access to data from hospitals and clinics is usually cited as a major hurdle to overcome for stakeholders seeking to conduct RWD studies (Cave et al., 2019; Rudrapatna and Butte, 2020; Grimberg et al., 2021), the clinicians employed by those institutions are willing to deliver those data directly or to at least allow them to be analyzed, under certain conditions. Here again, it should be highlighted that investigators belonging to the EORTC network are in all likelihood more inclined to contribute to research, so this observation may not extend to oncologists in general. Nonetheless, patients seem to display similar attitudes towards data sharing (Kalkman et al., 2022). Our results implicitly suggest that there is support on the part of many cancer physicians for initiatives such as the EMA’s Data Analysis and Real World Interrogation Network (DARWIN EU) (European Medicines Agency, 2022b), which will leverage RWD from across Europe to inform regulatory decision-making through the creation of a distributed network that operates based on a model of federated data access. As such, DARWIN EU will be the platform through which the EMA and the national medicines regulators will connect and interact with the European Health Data Space (European Commission, 2022). Eventually, DARWIN EU may also be used by payers and HTA bodies to answer questions relating to the cost-effectiveness of specific health technologies.

While RWE is considered to be complementary to evidence derived from RCTs (Eichler et al., 2020), RWD studies and RCTs are regularly juxtaposed against each other, especially in situations where they tackle comparable research questions but produce conflicting results (Hemkens et al., 2016; Franklin et al., 2021). Overall, even though our participants had positive views of RWE, they clearly depended more heavily on RCT-generated data than on RWD for their decision-making, and they expected other stakeholders such as regulators and payers to do the same. This is not a surprising finding, given that RCTs rank higher in the hierarchy of evidence (Oxford Centre for Evidence-Based Medicine, 2009) than any type of observational study design due to their reliance on randomization, which inherently reduces the risk of getting biased outcomes (Collins et al., 2020). In fact, respondents saw the methodological weaknesses of RWD studies as the most significant barrier to the use of RWE. However, not all studies relying on the analysis of RWD are burdened by the limitations of observational research: in pragmatic trials for instance, the allocation of the investigational therapies can be randomized, so there is no intrinsic need to adjust for confounders (Zuidgeest et al., 2017). Pragmatic trials are interventional studies that measure a treatment’s effectiveness (i.e. how well it works when applied in clinical practice, under real-life circumstances), rather than its efficacy (i.e. how well it works when applied in a highly controlled environment, under ideal circumstances) (Schwartz and Lellouch, 1967; Mullins et al., 2014; Ford and Norrie, 2016; Zuidgeest et al., 2017). They accomplish this by employing broadly formulated eligibility criteria to guide the recruitment of participants, by maximizing the level of flexibility given to investigators with respect to the administration of the intervention(s), and by minimizing the intensity with which trial subjects are monitored and followed up (Loudon et al., 2015). Of all the different RWD study designs our respondents were presented with, they thought pragmatic trials gave rise to the strongest RWE. From the answers we received to our survey, it is apparent that RCTs remain the principal source of evidence upon which cancer clinicians base their decisions, and that the methodological constraints of RWD studies can undermine the applicability of RWE in the clinic. More robust and actionable RWE can be obtained through the conduct of pragmatic trials, which combine the strengths of both RCTs and RWD studies (Zuidgeest et al., 2017). Nevertheless, in some situations, it may not be practically or ethically feasible to undertake RCTs. Tumor-agnostic agents for instance are standardly tested in non-randomized, biomarker-driven enrichment trials (e.g. basket studies) (Flaherty et al., 2017; Offin et al., 2018; du Rusquec and Le Tourneau, 2021; Seligson et al., 2021), which usually feature relatively small sample sizes. Here, observational RWD can offer valuable insights into the performance of these products in larger groups of patients (Agarwala et al., 2018; Miksad et al., 2019), as long as the quality of the data is sufficiently high. Since RWD of this type originate from routine clinical practice and not from research activities, they can be incomplete, inconsistent and subject to misclassification bias (Blacketer et al., 2021; Grimberg et al., 2021). Consequently, it is essential to evaluate the completeness, conformance and plausibility of the data by performing quality checks through the application of appropriate tools (Kahn et al., 2016; Blacketer et al., 2021).

RWD are typically collected and analyzed for the purpose of answering outstanding questions with regard to the safety and effectiveness of marketed medicines. Although our respondents believed that such data are suitable for tackling many of the uncertainties that accompany the adoption of novel anticancer treatments into the therapeutic armamentarium, they were not convinced that RWE can fully address all the evidence gaps they are confronted with in their daily work. For example, participants found RWD less useful for determining the optimal dose of antineoplastic drugs and for assessing their effectiveness relative to alternative interventions. It is likely that some uncertainties can only be conclusively resolved with the help of RCT-derived data (Lacombe et al., 2019a, 2019b; Saesen et al., 2020, 2021), which are often difficult to generate in the post-marketing setting. The EORTC has proposed a restructuring of the current framework for developing antitumor therapies so that the evidence gaps that will be encountered by downstream decision-makers such as payers and clinicians are identified and characterized at an early stage, allowing the necessary RCTs to be initiated as soon as possible, potentially even prior to the regulatory approval (Kempf et al., 2017; Lacombe et al., 2019b; EORTC, 2020). A paradigm shift of this nature would require support from all stakeholders, including regulators and policymakers. The EMA has recently established an academia-oriented forum in partnership with the EORTC that will explore how the use of cancer medicines in clinical practice can be optimized (European Medicines Agency, 2022c; Saesen et al., 2022).

This study suffers from a number of limitations. Firstly, the methodology used did not allow for an in-depth analysis of the reasons why the respondents selected specific response options. To investigate the motivations behind participants’ answers, additional questions of an open-ended nature would have needed to be included. This was considered impractical as it would have rendered the survey more burdensome and time-consuming to fill out, which in turn would have likely resulted in fewer respondents completing it. Alternatively, follow-up interviews could have been conducted with the participants, but this would have required the collection of their contact details, thereby compromising the confidentiality of their responses and reducing their willingness to truthfully convey their more controversial opinions.

Secondly, the questionnaire was exclusively sent out to European and Israeli clinicians who are part of the EORTC network. This implies that the survey results should not necessarily be extrapolated to oncologists who are not affiliated with this organization or who are working outside of Europe or Israel. This also means that all of the respondents had experience working as clinical trial investigators, which may have had an impact on their sentiments towards RCTs, since their academic careers could have been built on their involvement in studies of this kind.

Thirdly, as a result of the heterogeneity of the target population, some questions had to be phrased in such a way that all participants, regardless of their oncological expertise, would interpret them similarly, which made it difficult to present them with concrete scenarios or cases in which RWE has been or can be used. For instance, RWE was probably of greater importance to the respondents who were treating rare cancer patients, given that it is more challenging to perform RCTs in neoplasms that occur infrequently. This level of specificity could not be attained in this study.

Fourthly, although the questionnaire was distributed to clinicians from many different European countries, those from Western and Southern Europe were overrepresented in the survey sample. Their views may differ significantly from those of their colleagues in Northern and Eastern Europe, who did not participate in large numbers.

Fifthly, although we explained the differences between RWD and RWE to participants, we did not provide them with a general definition or description of these concepts. This was a deliberate choice we made in light of our intention to investigate their understanding of these terms. However, our observation that respondents interpreted RWD differently suggests that the survey questions may not have been understood in the same way by all clinicians.

Lastly, because this is a survey study, it may have been affected by various biases that have been described for questionnaire-based research. For example, even though reminder mails were sent out on multiple occasions, only 22.2% of the individuals that received an invitation to participate in the survey actually provided answers to the questions, which could signify that there was nonresponse bias. Additionally, participants may not have responded in a manner that reflects their true beliefs, potentially giving rise to response biases such as acquiescence bias or order effects bias. While the impact of the former was mitigated by including “no opinion” and “I don’t know” response options wherever possible, the latter could not be adequately addressed, as randomizing the order in which the questions appeared to respondents would have undermined the logical structure of the questionnaire.

## Conclusion

In conclusion, the cancer clinicians participating in this survey expressed favorable opinions about RWE and showed willingness to contribute to its generation, for example by sharing anonymized or pseudonymized electronic health data of their patients with fellow researchers. Depending on the source of the underlying RWD and the design of the study that delivers the evidence, they thought RWE can be relatively strong, although they also believed the methodological challenges accompanying its interpretation are difficult to overcome. They expected RWE to play an increasingly important role in the development of anticancer treatments in the future, and they saw this as a positive evolution. Nevertheless, they still viewed RCTs as the gold standard for evaluating the relationships between antineoplastic therapies and patient outcomes, and they did not consider RWE to be capable of fully addressing the knowledge gaps that remain after market entry of new antitumor interventions. Furthermore, for their decision-making, they continue to rely more heavily on RCT-derived evidence than on RWE. The results of this study suggest that there is fertile ground for involving European and Israeli clinicians in initiatives focusing on harnessing the evidentiary potential of RWD in the field of oncology, as long as such endeavors are appropriately balanced with the conduct of RCTs.

## Data Availability

The raw data supporting the conclusions of this article will be made available by the authors, without undue reservation.

## Footnotes

1 Please note that whenever this manuscript mentions “drug development,” “treatment development,” or variations of these terms, it assumes a broad perspective, referring not only to research carried out in the pre-approval setting (namely preclinical and phase I, II and III clinical studies), but also to post-marketing activities with relevance to the market access of therapies and their adoption in the clinic (e.g. phase IV clinical studies).
