# Supplementary material for: How do cancer clinicians perceive real-world data and the evidence derived therefrom? Findings from an international survey of the European Organisation for Research and Treatment of Cancer
Source: Front Pharmacol. 2022 Aug 24;13:969778. doi: 10.3389/fphar.2022.969778 (PMC9449152; doi:10.3389/fphar.2022.969778)
Supplement: Supplementary file 1 [file DataSheet1.DOCX]

**Supplementary materials: survey questions**

**PART 1: DEMOGRAPHICS**

We would like to collect your answers to the questions below to learn more about you. The responses to these questions will be used to describe the characteristics of the survey sample in reports and publications following from this study.

1. In which country do you currently live?

[List of countries to select from]

1. What is/are your field(s) of oncological expertise? (multiple options possible)

Brain cancers

Breast cancers

Endocrine cancers

Gastrointestinal cancers

Genitourinary cancers

Gynaecological cancers

Haematological cancers

Head and neck cancers

Lung cancers

Skin cancers

Soft tissue and bone cancers

Other (please specify)

1. What type(s) of therapies do you use to treat the cancer patients that you see in your daily practice? (multiple options possible)

Surgery

Radiotherapy

Chemotherapy

Immunotherapy

Hormonal therapy

Targeted therapy

Stem cell or bone marrow transplants

Other (please specify)

1. How many years have you been working as a clinician (rounding to the nearest integer)?

Between 0 and 5 years

Between 6 and 10 years

Between 11 and 15 years

Between 16 and 20 years

More than 20 years

1. What type of institution are you employed at? (multiple options possible)

University hospital

Community hospital

Private hospital

(Comprehensive) cancer center

Other (please specify)

**PART 2: UNDERSTANDING OF REAL-WORLD EVIDENCE AND ITS CURRENT PLACE IN THE TREATMENT DEVELOPMENT PARADIGM**

PLEASE NOTE: Throughout this survey, the terms *real-world evidence* and *real-world data* will be used as two closely related but distinct concepts. To clarify, *real-world evidence* is considered to be the evidence derived from the analysis of *real-world data*. As such, the difference between the two terms is equivalent to the difference between the broader concepts of *evidence* and *data*, i.e. *evidence* for or against a certain hypothesis is produced by analyzing *data*, and *data* in themselves do not constitute *evidence* prior to being interpreted by an intermediary actor.

1. Generally speaking, how do you view the evidence required within the current framework employed by regulatory authorities in your country or region for allowing new anticancer treatments to come to the market?

The current framework uses evidentiary standards that are too strict.

The current framework uses evidentiary standards that are too lenient.

The current framework uses the right evidentiary standards.

No opinion.

1. In your view, which of the following aspects is most important to consider during the regulatory approval process of a new anticancer treatment?

The need for rapid patient access.

The need for robust and mature evidence.

Both are equally important.

No opinion.

1. To which extent do you (dis)agree with the following statements?
   1. Once a new cancer therapy has entered the market, there is typically sufficient information available to guide clinicians on how it should be used in clinical practice.

Strongly agree

Agree

Neither agree nor disagree

Disagree

Strongly disagree

- 1. The current framework employed by regulatory authorities within my country or region for allowing new cancer therapies to come to the market is in need of reform.

Strongly agree

Agree

Neither agree nor disagree

Disagree

Strongly disagree

- 1. Randomized controlled trials (RCTs) should still be considered the gold standard for generating clinical evidence in the field of oncology.

Strongly agree

Agree

Neither agree nor disagree

Disagree

Strongly disagree

- 1. Real-world evidence cannot fully address the evidence gaps that currently remain once a new anticancer treatment has entered the market.

Strongly agree

Agree

Neither agree nor disagree

Disagree

Strongly disagree

- 1. Real-world evidence is a well-defined concept.

Strongly agree

Agree

Neither agree nor disagree

Disagree

Strongly disagree

1. Which of the following potential definitions of real-world data corresponds the most with your understanding of this concept?

Data collected in a non-experimental setting (i.e. health data collected without prior intent of being used for research purposes)

Data collected in a non-interventional/non-controlled setting (i.e. health data collected without influencing the conditions under which they are generated)

Data collected in a non-RCT setting (i.e. health data collected outside of randomized controlled trials)

None of the above

1. What connotation, if any, does the term real-world evidence have for you?

| Very negative | Moderately negative | Slightly negative | Neutral | Slightly positive | Moderately positive | Very positive |
| --- | --- | --- | --- | --- | --- | --- |
|  |  |  |  |  |  |  |

**PART 3: USE AND VALUE OF REAL-WORLD EVIDENCE**

1. How would you evaluate the strength of the evidence produced by the following study designs that are capable of generating real-world evidence? You can consult their definitions by clicking on them.
   1. Case-control studies [Pop-up definition: An observational study design that compares patients who have a disease or outcome of interest (cases) with patients who do not have the disease or outcome (controls), and looks back retrospectively to compare how frequently the exposure to a risk factor is present in each group to determine the relationship between the risk factor and the disease.]

| Very weak | Rather weak | Moderate | Rather strong | Very strong |
| --- | --- | --- | --- | --- |
|  |  |  |  |  |

No opinion.

- 1. Cohort studies [Pop-up definition: An observational study design where one or more samples (called cohorts) are followed prospectively and subsequent status evaluations with respect to a disease or outcome of interest are conducted to determine which prior exposure characteristics (risk factors) are associated with it.]

| Very weak | Rather weak | Moderate | Rather strong | Very strong |
| --- | --- | --- | --- | --- |
|  |  |  |  |  |

No opinion.

- 1. Cross-sectional studies [Pop-up definition: An observational study design which examines the relationship between a disease or a specific outcome and certain variables of interest as they exist in a defined population at a single point in time or over a short period of time.]

| Very weak | Rather weak | Moderate | Rather strong | Very strong |
| --- | --- | --- | --- | --- |
|  |  |  |  |  |

No opinion.

- 1. Pragmatic trials [Pop-up definition: An interventional study design which compares several health interventions among a randomized, diverse population representing clinical practice and measures their real-world effectiveness.]

| Very weak | Rather weak | Moderate | Rather strong | Very strong |
| --- | --- | --- | --- | --- |
|  |  |  |  |  |

No opinion.

1. How would you evaluate the strength of the evidence produced by studies relying on the following real-world data sources? You can consult their definitions by clicking on them.
   1. Patient registries [Pop-up definition: Patient registries are organized systems that use observational methods to collect uniform data on specified outcomes in a population defined by a particular disease, condition or exposure.]

| Very weak | Rather weak | Moderate | Rather strong | Very strong |
| --- | --- | --- | --- | --- |
|  |  |  |  |  |

No opinion.

- 1. Electronic health records [Pop-up definition: Electronic health records are digital versions of patients’ health records, documenting clinical and administrative encounters between patients and healthcare providers.]

| Very weak | Rather weak | Moderate | Rather strong | Very strong |
| --- | --- | --- | --- | --- |
|  |  |  |  |  |

No opinion.

- 1. Administrative claims [Pop-up definition: Administrative claims databases are database systems built on data extracted from claims that are submitted by healthcare providers to payers when a patient uses health services. They include pharmacy databases and health insurance records.]

| Very weak | Rather weak | Moderate | Rather strong | Very strong |
| --- | --- | --- | --- | --- |
|  |  |  |  |  |

No opinion.

- 1. Social media [Pop-up definition: Social media are online platforms allowing people to connect with other people and share information about themselves. Examples include Facebook, Twitter, LinkedIn and Instagram.]

| Very weak | Rather weak | Moderate | Rather strong | Very strong |
| --- | --- | --- | --- | --- |
|  |  |  |  |  |

No opinion.

PLEASE NOTE: For the remaining questions, when the term randomized controlled trial (RCT) is used, pragmatic trials are left out of consideration due to their unique characteristics.

1. What role do you think real-world evidence (RWE) should play in the decision-making process of regulatory authorities concerning the regulatory approval of new anticancer treatments compared with RCT-derived evidence?

| RWE should not be considered at all | A much smaller role | A somewhat smaller role | An equal role | A somewhat larger role | A much larger role | No evidence other than RWE should be considered |
| --- | --- | --- | --- | --- | --- | --- |
|  |  |  |  |  |  |  |

No opinion.

1. What role do you think real-world evidence (RWE) should play in the decision-making process of payers concerning the reimbursement of new anticancer treatments compared with RCT-derived evidence?

| RWE should not be considered at all | A much smaller role | A somewhat smaller role | An equal role | A somewhat larger role | A much larger role | No evidence other than RWE should be considered |
| --- | --- | --- | --- | --- | --- | --- |
|  |  |  |  |  |  |  |

No opinion.

1. What role does real-world evidence (RWE) play in your decision-making process concerning the treatments you administer to your patients compared with RCT-derived evidence?

| I do not rely on RWE at all | A much smaller role | A somewhat smaller role | An equal role | A somewhat larger role | A much larger role | I only rely on RWE |
| --- | --- | --- | --- | --- | --- | --- |
|  |  |  |  |  |  |  |

No opinion.

1. If an RCT concluded that a particular anticancer treatment resulted in a significant improvement in survival over the standard of care but a subsequent study based on the analysis of real-world data came to the conclusion that there is no survival benefit, which of the two would you be more inclined to trust?

The RCT

The study based on real-world data

Depends on the context

No opinion

1. In the scenario of question 16, do you think such real-world evidence should be able to lead to a change in the marketing authorization (e.g. a retraction of an approved indication or of the approval altogether)?

Yes

No

Depends on the context

No opinion

1. In the scenario of question 16, do you think such real-world evidence should be able to lead to a change in the reimbursement conditions (e.g. a reduction in coverage or a retraction of the reimbursement altogether)?

Yes

No

Depends on the context

No opinion

1. If regulatory authorities approve an anticancer treatment on the condition that uncertainties regarding its effectiveness are addressed in the post-approval setting, which type of studies should ideally be performed to satisfy this requirement according to you?

Clinical trials

Studies based on real-world data

A combination of both clinical trials and studies based on real-world data

No opinion

1. To what extent do you think the following challenges that have been described in the literature relating to the collection and use of real-world data can be overcome?
   1. Operational challenges (i.e. challenges relating to data access, data

protection, data sharing, etc.)

| No issue at all | Easy to overcome | Can be overcome with some effort | Difficult to overcome | Cannot be overcome |
| --- | --- | --- | --- | --- |
|  |  |  |  |  |

No opinion.

- 1. Technical challenges (i.e. challenges relating to data formatting, data

validation, data linkage, etc.)

| No issue at all | Easy to overcome | Can be overcome with some effort | Difficult to overcome | Cannot be overcome |
| --- | --- | --- | --- | --- |
|  |  |  |  |  |

No opinion.

- 1. Methodological challenges (i.e. challenges relating to missing data,

selection biases, confounding factors, statistical analyses, etc.)

| No issue at all | Easy to overcome | Can be overcome with some effort | Difficult to overcome | Cannot be overcome |
| --- | --- | --- | --- | --- |
|  |  |  |  |  |

No opinion.

1. To what extent do you think real-world data is suitable for tackling the following uncertainties relating to the adoption of new anticancer treatments into clinical practice once they have entered the market?
   1. Uncertainties relating to their safety:

| Highly unsuitable | Unsuitable | Neither suitable nor unsuitable | Suitable | Highly suitable |
| --- | --- | --- | --- | --- |
|  |  |  |  |  |

No opinion.

- 1. Uncertainties relating to their (comparative) effectiveness:

| Highly unsuitable | Unsuitable | Neither suitable nor unsuitable | Suitable | Highly suitable |
| --- | --- | --- | --- | --- |
|  |  |  |  |  |

No opinion.

- 1. Uncertainties relating to their costs and economic impact:

| Highly unsuitable | Unsuitable | Neither suitable nor unsuitable | Suitable | Highly suitable |
| --- | --- | --- | --- | --- |
|  |  |  |  |  |

No opinion.

- 1. Uncertainties relating to their optimal dosing:

| Highly unsuitable | Unsuitable | Neither suitable nor unsuitable | Suitable | Highly suitable |
| --- | --- | --- | --- | --- |
|  |  |  |  |  |

No opinion.

- 1. Uncertainties relating to their optimal duration of treatment:

| Highly unsuitable | Unsuitable | Neither suitable nor unsuitable | Suitable | Highly suitable |
| --- | --- | --- | --- | --- |
|  |  |  |  |  |

No opinion.

- 1. Uncertainties relating to their combination and sequencing with other

therapies:

| Highly unsuitable | Unsuitable | Neither suitable nor unsuitable | Suitable | Highly suitable |
| --- | --- | --- | --- | --- |
|  |  |  |  |  |

No opinion.

- 1. Uncertainties relating to their effects on quality of life:

| Highly unsuitable | Unsuitable | Neither suitable nor unsuitable | Suitable | Highly suitable |
| --- | --- | --- | --- | --- |
|  |  |  |  |  |

No opinion.

- 1. Uncertainties relating to their optimal target population of patients:

| Highly unsuitable | Unsuitable | Neither suitable nor unsuitable | Suitable | Highly suitable |
| --- | --- | --- | --- | --- |
|  |  |  |  |  |

No opinion.

1. How do you evaluate the importance of real-world data within the context of the following activities:
   1. Diagnosis of patients:

| Very low importance | Low importance | Moderate importance | High importance | Very high importance |
| --- | --- | --- | --- | --- |
|  |  |  |  |  |

No opinion.

- 1. Monitoring of patients:

| Very low importance | Low importance | Moderate importance | High importance | Very high importance |
| --- | --- | --- | --- | --- |
|  |  |  |  |  |

No opinion.

- 1. Making treatment decisions for patients:

| Very low importance | Low importance | Moderate importance | High importance | Very high importance |
| --- | --- | --- | --- | --- |
|  |  |  |  |  |

No opinion.

**PART 4: EXPERIENCE WITH REAL-WORLD EVIDENCE AND FUTURE EVOLUTIONS**

1. To your knowledge, are there any studies based on the collection of real-world data currently ongoing at your hospital or institution?

Yes

No

I don’t know

1. Have you personally been involved in any studies relying on the analysis of real-world data?

Yes, I have.

No, I have not, and I would not be interested in getting involved in such studies in the future.

No, I have not, but I would be interested in getting involved in such studies in the future.

I don’t know

1. Would you support the transfer of anonymized or pseudonymized electronic health data of your patients into a central international repository that can be used by others exclusively for research purposes?

Yes

No

Maybe, depending on the context

No opinion

1. Would you support having anonymized or pseudonymized electronic health data of your patients made accessible to others inside or outside your country exclusively for research purposes, without these data being transferred from your hospital or institution to an external repository?

Yes

No

Maybe, depending on the context

No opinion

1. How do you see the role of real-world evidence in the development of anticancer treatments evolve in the future?

It will become increasingly important over time.

It will become less important over time.

Its importance will remain stable over time.

No opinion.

1. If real-world evidence were to play an increasingly important role in the development of anticancer treatments in the future, how would you perceive this trend?

Positively.

Neutrally.

Negatively.

No opinion.

**PART 5: ENDING**

Thank you for your participation to this study. Your input is valued and will contribute to our understanding about the use of real-world evidence in oncology.

If you have any further comments related to this study, or related to the broader context of this topic, please feel free to include them in the text box below:

[Type your remarks here]
